# The Spectrum of SWI/SNF Mutations, Ubiquitous in Human Cancers

**DOI:** 10.1371/journal.pone.0055119

**Published:** 2013-01-23

**Authors:** A. Hunter Shain, Jonathan R. Pollack

**Affiliations:** Department of Pathology, Stanford University School of Medicine, Stanford, California, United States of America; George Mason University, United States of America

## Abstract

SWI/SNF is a multi-subunit chromatin remodeling complex that uses the energy of ATP hydrolysis to reposition nucleosomes, thereby modulating gene expression. Accumulating evidence suggests that SWI/SNF functions as a tumor suppressor in some cancers. However, the spectrum of SWI/SNF mutations across human cancers has not been systematically investigated. Here, we mined whole-exome sequencing data from 24 published studies representing 669 cases from 18 neoplastic diagnoses. SWI/SNF mutations were widespread across diverse human cancers, with an excess of deleterious mutations, and an overall frequency approaching *TP53* mutation. Mutations occurred most commonly in the *SMARCA4* enzymatic subunit, and in subunits thought to confer functional specificity (*ARID1A*, *ARID1B*, *PBRM1*, and *ARID2*). SWI/SNF mutations were not mutually-exclusive of other mutated cancer genes, including *TP53* and *EZH2* (both previously linked to SWI/SNF). Our findings implicate SWI/SNF as an important but under-recognized tumor suppressor in diverse human cancers, and provide a key resource to guide future investigations.

## Introduction

SWitch/Sucrose NonFermentable (SWI/SNF) is a chromatin remodeling complex originally identified in yeast genetic screens for yeast mating-type switching and sucrose fermentation genes [1], [2]. These seemingly disparate activities underscore its wide-ranging roles in diverse biological processes. SWI/SNF uses the energy of ATP hydrolysis to reposition nucleosomes, thus regulating access to the DNA and modulating transcription and DNA replication/repair [3].

The SWI/SNF complex, conserved from yeast to humans, is composed of 10–15 biochemically-distinct subunits (reviewed here [3]–[5]). In humans the complex contains either of two mutually-exclusive ATPase enzymatic subunits, SMARCA2 (hBRM) or SMARCA4 (BRG). In addition, the complex includes either of three mutually-exclusive subunits thought to confer functional specificity: ARID1A (BAF250A), ARID1B (BAF250B), or PBRM1 (BAF180). ARID1A and ARID1B are found associated with “BAF” complexes (BRG1- or hBRM-associated factors), which contain either enzymatic subunit. PBRM1, together with ARID2 (BAF200) and BRD7, is found only in “PBAF” complexes (polybromo-associated BAF), which contain SMARCA4. Lastly, there are several “core” and “accessory” subunits (some mutually exclusive) that are associated with all versions of the complex: SMARCB1 (BAF47/SNF5), SMARCC1 (BAF155), SMARCC2 (BAF170), SMARCE1 (BAF57), [SMARCD1 (BAF60A), SMARCD2 (BAF60B), or SMARCD3 (BAF60C)], [PHF10 (BAF45A), DPF1 (BAF45B), or DPF2 (BAF45D)]; DPF3 (BAF45C); and [ACTL6A (BAF53A) or ACTL6B (BAF53B)]. The various combinatorial assemblies are thought to support context-dependent activities of the complex.

Over the past decade, evidence has mounted to indicate that SWI/SNF plays a tumor suppressive role in human cancer – thoroughly reviewed elsewhere [3]–[5]. The most compelling case has been that of *SMARCB1* (SNF5), which was discovered to be homozygously inactivated in nearly all rhabdoid tumors (a rare pediatric malignancy) [6]. Follow-up studies revealed that *SMARCB1* knockout mice are prone to similar tumors [7]. Subsequent studies reported mutations that implicated other SWI/SNF subunits, including *SMARCB1* in lung cancer [8], [9]. However, despite these studies, the role of SWI/SNF complexes in cancer had gone largely underappreciated for many years.

Recently, with the advent of fast and cheaper DNA sequencing technologies, whole-exome surveys of human cancers have re-invigorated mutation discovery efforts. Several such studies have newly reported components of SWI/SNF to be mutated at high frequency in single cancer types, garnering renewed excitement surrounding SWI/SNF and cancer [10]–[16]. However, most published exome studies have focused only on “top hits”, as those most-highly mutated genes. There has been no systematic effort to define the frequency and spectrum of SWI/SNF subunit mutations across human cancers. Thus, here we report the mutational spectrum of 20 canonical SWI/SNF subunit genes across 18 different cancer diagnoses, drawing from 24 whole-exome sequencing studies representing 669 patient samples. The resultant macroscopic view of the complex affords unique insights into the genetics and tumor biology of SWI/SNF.

**Figure 1:**
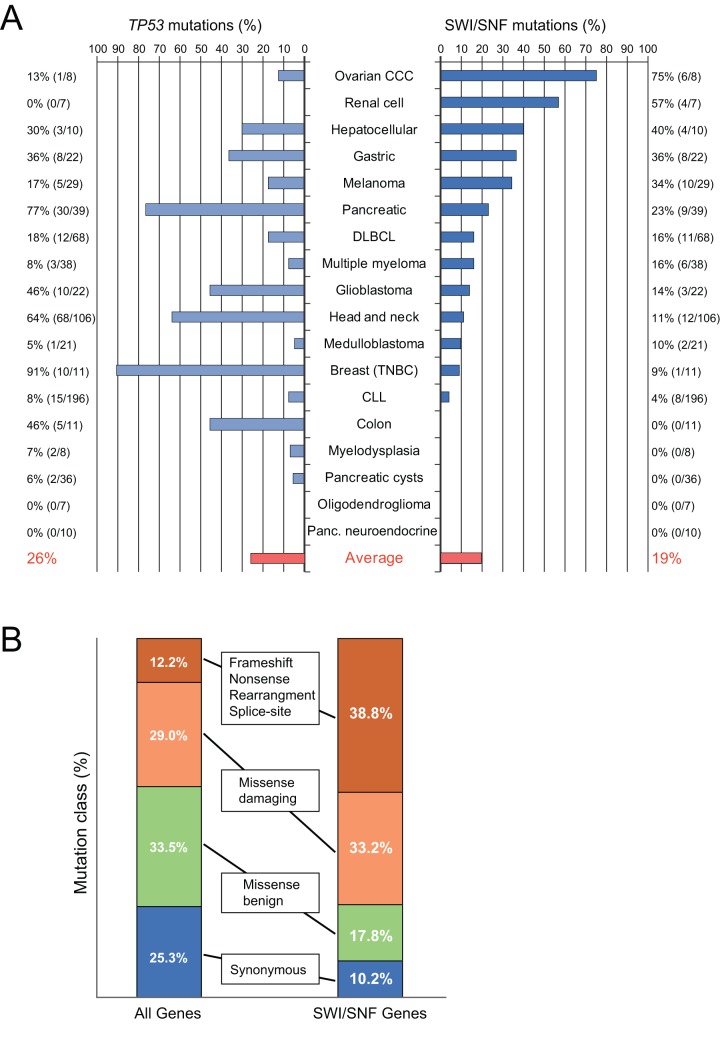
SWI/SNF mutations are deleterious and widespread across human cancers. **A.** Bar graph depicts the frequency of nonsynonymous mutations in SWI/SNF (*right*; counting mutations in any of 20 subunit genes) and *TP53* (*left*) for each of the 18 tumor diagnoses surveyed. The average frequency of the 18 tumor diagnoses is indicated in red. The small number of samples with mutations in two different SWI/SNF subunits was not double-counted. **B.** The frequency distribution by mutation class is indicated for SWI/SNF subunit genes (*right*) and for all exome-sequenced genes (*left*). Note, the class distribution of SWI/SNF mutations is significantly skewed towards deleterious mutations (*P* = 1.0×10^−18^, chi-square test). Refer to Methods for a detailed description of these data.

Remarkably, mutations in SWI/SNF were present at high frequency across many different tumor types (Fig. 1A). The cancers with the highest SWI/SNF mutation rates were ovarian clear cell carcinoma (75%), clear cell renal cell carcinoma (57%), hepatocellular carcinoma (40%), gastric cancer (36%), melanoma (34%), and pancreatic cancer (26%). Across all tumor types, the average frequency of SWI/SNF mutations (19%) approached that of *TP53* (26%; shown for comparison in Fig. 1A), the single-most mutated tumor suppressor gene.

## Results and Discussion

### SWI/SNF mutations are common across diverse cancer types

To survey the spectrum of SWI/SNF mutations in human cancers, we analyzed data from 24 whole-exome studies [10]–[13], [17]–[36] together spanning 18 diverse cancer types (see Methods). Selected characteristics of the 24 studies are summarized in Table 1. More detailed information, including characteristics of the sequencing platform, fold-sequencing coverage, and genome-wide mutation frequencies (by mutation type and predicted impact) are summarized in Table S1. The mutational status of the 20 genes encoding canonical subunits of human SWI/SNF is detailed in Table S2.

**Table 1:** Exome studies analyzed.

Study	Citation	Sample size	Cancer type(s)
1	Wood et al. [17]	22	Breast (triple negative) (n = 11); Colon (n = 11)
2	Quesada et al. [18]	105	Chronic lymphocytic leukemia
3	Wang et al. [19]	91	Chronic lymphocytic leukemia
4	Lohr et al. [20]	49	Diffuse large B-cell lymphoma
5	Pasqualucci et al. [21]	6	Diffuse large B-cell lymphoma
6	Morin et al. [22]	13	Diffuse large B-cell lymphoma
7	Wang et al. [13]	22	Gastric
8	Parsons et al. [23]	22	Glioblastoma
9	Stransky et al. [24]	74	Head & Neck
10	Agrawal et al. [25]	32	Head & Neck
11	Li et al. [11]	10	Hepatocellular
12	Parsons et al. [26]	21	Medulloblastoma
13	Wei et al. [27]	14	Melanoma
14	Nikolaev et al. [28]	7	Melanoma
15	Stark et al. [29]	8	Melanoma
16	Chapman et al. [30]	38	Multiple Myeloma
17	Yoshida et al. [31]	28	Myelodysplasia
18	Bettegowda et al. [32]	7	Oligodendroglioma
19	Jones et al. [10]	8	Ovarian Clear Cell Carcinoma
20	Jones et al. [33]	24	Pancreatic
21	Wang et al. [34]	15	Pancreatic
22	Wu et al. [35]	32	Pancreatic Cyst
23	Jiao et al. [36]	10	Pancreatic Neuroendocrine
24	Varela et al. [12]	7	Renal Cell Carcinoma (clear cell)

Given the size of the SWI/SNF genomic “footprint” (spanning 20 genes), it might be argued that SWI/SNF is prone to passenger mutations that could inflate the mutational frequency of the complex. To address this concern, we compared the distribution of mutation types in SWI/SNF subunit genes to that of the whole exome (Fig. 1B). Our analysis revealed a notable skew of mutations in SWI/SNF genes, with a significantly increased fraction of predicted deleterious mutations (frameshift, nonsense, rearrangement, splice-site, and missense-damaging) compared to predicted missense-benign and synonymous mutations (*P* = 1.0×10^−18^, chi-square test). This pattern suggests that most observed SWI/SNF mutations are likely driver alterations.

Indeed, rather than overestimating the frequency of SWI/SNF inactivation (due to some level of passenger mutations), the sequencing analysis likely underestimates the true frequency of SWI/SNF inactivation. Evidence exists that genomic DNA deletions, rearrangements, and epigenetic silencing provide alternative mechanisms to inactivate SWI/SNF subunits [14], [37]. Moreover, the impact of mutations on protein expression and function has not been adequately explored. Only one of the exome studies analyzed here also evaluated protein levels, finding *ARID1A* mutations associated with reduced or lost ARID1A expression (by immunohistochemistry) in gastric cancer [13] (and the same had been separately shown for ovarian clear cell carcinoma [15]). Systematic efforts to survey all genetic, epigenetic and protein changes would be needed to arrive at the true frequency of SWI/SNF alterations.

### SWI/SNF mutations in specific cancer types

Due to its high mutation frequency (36–75%; Fig. 1A), a likely tumor suppressive role of the SWI/SNF complex had been recognized by the respective study authors in ovarian clear cell carcinoma, clear-cell renal cell carcinoma, hepatocellular carcinoma, gastric cancer, and pancreatic cancer [10]–[16]. Nonetheless, nearly all of those studies highlighted only a single highly-mutated subunit (e.g. *ARID1A* mutation in ovarian clear cell carcinoma), whilst our analysis also uncovered less frequent mutations of other SWI/SNF subunits in those same tumor types (Table S2).

Notably, the mutational data implicating a tumor suppressive role of SWI/SNF (mutation frequencies 11–34%; Fig. 1A) are also compelling for melanoma, diffuse large B-cell lymphoma (DLBCL), multiple myeloma, glioblastoma, and head and neck cancers, but had not been appreciated. In these cancers, SWI/SNF mutations likely went unnoticed because they were spread across multiple SWI/SNF subunits, none by itself reaching a critical threshold. Among this group of cancers, melanoma exhibited the highest SWI/SNF mutation rate.

While melanomas have an inherently high mutation rate from UV exposure, the mutations noted here display characteristics of tumor suppressor driver mutations. Among 29 cases sequenced [27]–[29], 17 nonsynonymous mutations struck *ARID1A* (n = 5), *SMARCA4* (n = 4), *ARID2* (n = 3), *SMARCB1* (n = 3), *SMARCA2* (n = 1), and *SMARCC1* (n = 1) (Table S2). These include a homozygous mutation in *ARID2* and three mutations targeting *SMARCB1* in the same patient sample, thus likely affecting both alleles. Furthermore, the mutation types included 5 nonsense mutations, 9 probably-damaging missense mutations (as called by polyphen-2 [38]), 1 possibly-damaging missense mutation, and 2 benign missense mutations. Only one of the three melanoma studies [28] reported on synonymous mutations, where there were 2 synonymous mutations (one each for *SMARCA4* and *SMARCC1*) compared to 7 nonsynonymous mutations (the nonsynonymous: synonymous mutation ratio in the melanoma exome was 1.9:1). Given the synonymous mutations, and the relatively high mutation rate in melanoma, there is likely some background passenger mutation rate of SWI/SNF in melanoma. Nonetheless, the loss of heterozygosity (LOH; implied by the homozygous mutation and multiple mutations in the same gene and sample), the damaging mutation skew, and the recurrence of mutations for several subunits altogether suggest that many or most of these mutations are drivers.

In 68 diffuse large B-cell lymphomas [20]–[22], nonsynonymous mutations targeted *ARID1B* (n = 4), *ARID1A* (n = 2), *PBRM1* (n = 2), *DPF2* (n = 2), *SMARCC2* (n = 1), and *SMARCD3* (n = 1). Those mutations can be classified into the following types: nonsense (n = 3), frameshift (n = 1), splice-site (n = 1), probably-damaging missense (n = 2), benign-missense (n = 1), and missense mutations of undetermined significance (n = 4). LOH information was not available from the two DLBCL studies. One study [20] reported on synonymous mutations, and only 1 synonymous mutation occurred in *ARID2* compared to 9 nonsynonymous mutations across several other SWI/SNF subunit genes. The high frequency of mutations, the recurrence within genes, and the deleterious skew of mutations all suggest a tumor suppressive role of SWI/SNF in DLBCL.

From 38 multiple myelomas that were sequenced [30], six had mutations in six different SWI/SNF subunits, broken down as follows: 2 rearrangements, 2 probably-damaging missense mutations, and 2 benign-missense mutations. The frequency of synonymous mutations and LOH information was not available from this study. Thus, the case for SWI/SNF as a tumor suppressor relies mostly on the frequency of SWI/SNF mutations. Notably, the background mutation rate was not particularly high for multiple myeloma, and consequently the SWI/SNF mutations are unlikely to all represent merely passenger events.

In glioblastoma multiforme (GBM) [23], 4 of 22 samples harbored SWI/SNF mutations. One sample had two different mutations in the same gene (*ARID1A*) suggesting mutations striking both alleles and making a strong case that those mutations are driver mutations. *SMARCA4*, *SMARCA2*, and *SMARCC2* each had a single probably-damaging missense mutation, though *SMARCC2* also had a synonymous mutation. Larger validation efforts will be necessary, but the overall mutational pattern is suggestive of driver mutations in GBM.

Head and neck cancers had a total of 12 mutations out of the 106 samples sequenced in two studies [24], [25]. The mutational frequency in head and neck cancers is relatively high due to tobacco exposure in a subset of patients. Nonetheless, the 12 mutations hitting *ARID1A*, *ARID1B*, *PBRM1*, *ARID2*, *SMARCA4*, *SMARCA2*, and *SMARCC2* can be broken down as follows: 3 nonsense, 1 frameshift, 4 probably-damaging missense, 2 possibly-damaging missense, and 2 benign-missense mutations, representing a skew towards deleterious mutations relative to exome-wide mutational statistics. Some genes were recurrently mutated, including *ARID1A*, *ARID1B*, *SMARCA4*, and *PBRM1*. Information regarding LOH and synonymous mutations were not available from these studies.

Medulloblastoma, breast cancer, and chronic lymphocytic leukemia (CLL) all showed lower but likely significant SWI/SNF mutation rates (4–10%; Fig. 1A). In the case of medulloblastoma [26], there was a frameshift mutation in *ARID1A* and a possibly-damaging missense mutation in *SMARCA4*. In addition to the mutations from the full exome sequencing data, *SMARCA4* was found mutated in 2 additional samples from a validation cohort associated with the same study [26]. Furthermore, three *ARID1A* mutations have been reported in separate validation efforts [39].

As for breast cancer [17], only a single damaging missense mutation was identified in *ARID1B*. However, the sample set was small and not reflective of known breast cancer heterogeneity (all 11 samples from the study were triple negative breast cancer, i.e. negative for estrogen receptor, progesterone receptor and Her2); thus conclusions should be tempered. Nevertheless, other reports have identified *ARID1A* and *PBRM1* mutations in breast cancer [39]–[42], suggesting a likely tumor suppressive role of the complex.

In the case of CLL [18], [19], 4.5% of cases harbored SWI/SNF mutations. Although relatively low, this frequency is likely meaningful for several reasons. First, there were 196 CLL cases sequenced between the two studies, making this one of the higher powered cancer types included in this analysis, and 8 of these cases had a mutation in a SWI/SNF subunit. Two mutations each hit *ARID1A* and *BRD7*, suggesting some level of recurrence within subunits. Of the eight total mutations, 1 was a nonsense mutation, 5 were probably-damaging missense mutations, and 2 were predicted benign-missense mutations, again suggesting a skew towards damaging mutations (synonymous mutations were not reported in these two studies). Importantly, the overall mutation rate for CLL is relatively low; the average CLL case had only 15 mutations, corresponding to a mutation rate of less than 1 mutation/Mb of exome sequenced. Furthermore, the single-most mutated gene in CLL, *SF3B1*, was itself mutated in only 15% of cases. Thus, the observed SWI/SNF mutations, albeit uncommon, are likely meaningful.

No SWI/SNF mutations were identified in colon cancer, myelodysplasia, oligodendroglioma, pancreatic neuroendocrine tumors, and pancreatic cysts [17], [31], [32], [35], [36]. It is worth noting that these neoplasms, with the exception of colon cancer, tend to be less aggressive or even benign. However, it is possible that SWI/SNF mutations do occur but were not evident because the studies were under-powered, or because the sample sets were biased. In that regard, the colon cancers sequenced appear to all be microsatellite stable (based on the low overall mutation frequencies), and thus not representative of all colon cancer subtypes. Intriguingly, SWI/SNF mutations in gastric cancers tended to occur in microsatellite instable (MSI) tumors [13]. Indeed, targeted resequencing of *ARID1A* across several cancer types suggests that it is inactivated in a high fraction of MSI colon cancer cases [39]. Furthermore, *SMARCC1* has been reported to be deficient in a single colon cancer cell line [37]. Thus the evidence overall suggests that SWI/SNF may play a role in colon tumorigenesis.

The pancreatic cyst study [35] sequenced eight samples of serous cystadenomas (SCAs), intraductal papillary mucinous neoplasms (IPMNs), mucinous cystic neoplasms (MCNs), and solid pseudopapillary neoplasms (SPNs). IPMNs and MCNs are the only cysts with the capacity to evolve to frank adenocarcinoma; yet they are not the canonical pancreatic intraepithelial neoplasia (PanIN) lesions that typically precede pancreatic ductal adenocarcinoma (PDAC) [43]. In contrast to the exome data, loss of SMARCA4 expression at the protein level has been reported in a subset of IPMNs [44]. More work is needed to characterize the status of SWI/SNF in pancreatic cancer precursor lesions, and for that matter the timing of SWI/SNF mutations during the development and progression of other human cancers.

One remarkable finding of our analysis is the breadth of tumor types in which SWI/SNF is mutated – from brain, to hematopoietic, to various solid epithelial cancers. Albeit different tumor types exhibit different SWI/SNF mutation frequencies and subunit preferences (discussed more below), SWI/SNF mutations on the whole are not confined to any tissue origin, histologic, or molecular subtype of cancer. Mechanistically, this raises the possibility that SWI/SNF could work through a general tumor suppressor pathway(s) rather than any lineage-specific pathway.

### SWI/SNF mutations preferentially target certain subunits

**Figure 2:**
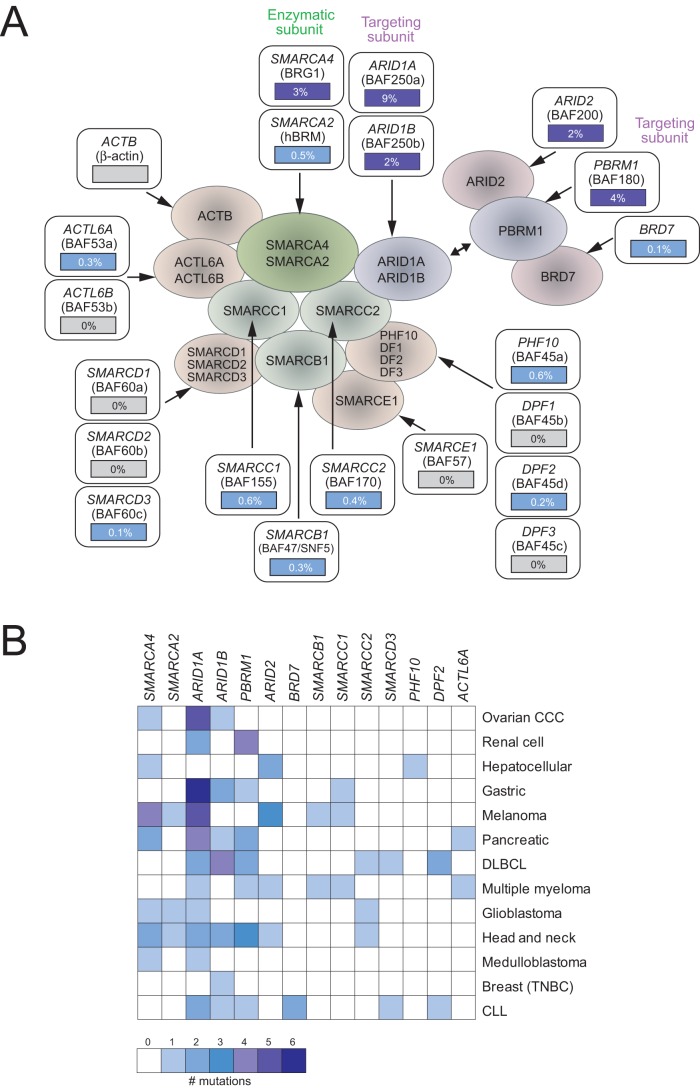
Some SWI/SNF subunits are preferentially mutated. **A.** The average frequency of nonsynonymous SWI/SNF subunit mutations (for the 18 tumor diagnoses analyzed) is indicated superimposed on a schematic depiction of the SWI/SNF complex. Mutations preferentially hit the *SMARCA4* enzymatic subunit and several targeting subunits (*ARID1A*, *ARID1B*, *PBRM1*, and *ARID2*). **B.** Heatmap (color scale indicated) depicting the number of nonsynonymous mutations found in each SWI/SNF subunit gene from the exome datasets analyzed. Note that some tumor types show selective mutation of single SWI/SNF subunits, e.g. *ARID1A* in ovarian clear cell carcinoma (CCC) and gastric cancer, while most other tumor types do not. For simplification, only those SWI/SNF subunits and tumor types having mutations are shown.

The above data indicate that SWI/SNF subunits are frequently mutated in human cancers. We next asked whether certain subunits in the complex were preferentially targeted. The SWI/SNF complex subunits can be assigned to roughly three functionally distinct groups – an enzymatic subunit (SMARCA2 or SMARCA4), a subunit thought to confer functional specificity to the complex (hereafter referred to as targeting subunits; ARID1A, ARID1B or PBRM1), and the remaining core and variant subunits (hereafter referred to as scaffolding subunits) [3]. Across the 13 cancer types with SWI/SNF mutations, the preponderance of mutations occurred in the SMARCA4 enzymatic subunit and in the three targeting subunits (Fig. 2A). Mutations did occur but less commonly in scaffolding subunits.

The finding that mutations occur across several different SWI/SNF subunits suggests that the main impact of mutations may be to compromise in part or whole the functional activity of the complex. The preponderance of mutations in the enzymatic and targeting subunits suggests that those subunits may be most critical to SWI/SNF function. Consistent with this interpretation, germline mutations of multiple SWI/SNF subunits were recently found to underlie Coffin-Siris syndrome (CSS; a rare developmental disorder) [45], implying a genetic equivalence of different subunits. Of 16 SWI/SNF subunits sequenced across 23 individuals with CSS, mutations were found in *SMARCA4* (26%), *ARID1B* (26%), *SMARCB1* (17%), *ARID1A* (13%), *SMARCA2* (4%), and *SMARCE1* (4%) [45]. Notably, the set of affected SWI/SNF subunits to a large extent mirrors that of human cancers, supporting that certain enzymatic and targeting subunits are likely most critical to the function of the complex. Nonetheless, there is likely to be additional subtlety with regard to possible distinct functions of SWI/SNF complexes with different subunit compositions, cell and tissue-type specificity of those complexes, and in the case of mutations, possible compensatory activity of residual SWI/SNF complexes (containing non-mutated alternative subunits).

Indeed, viewing each of the 13 cancer types separately, interesting mutational patterns emerge (Fig. 2B). Some cancer types exhibit mutations predominantly in a single SWI/SNF subunit, including (and as also noted by the authors in those studies) *ARID1A* in ovarian clear cell carcinoma and gastric cancer, and *PBRM1* in renal cell carcinoma. Most others, including melanoma, pancreatic cancer, and DLBCL, exhibit a more balanced spectrum of mutations among the commonly mutated subunits. For those tumor types where a single subunit is predominantly affected, it is possible that the subunit (and the complexes containing it) has cell or tissue-type specific functions that account for its selective inactivation. Such is almost certainly the case for the finding of *SMARCB1* (SNF5) mutations in all rhabdoid tumors [7]. Alternatively, cell or tissue-type specific mutational processes (e.g., relating to access of genomic loci) might be operating.

An interesting question is whether within any particular SWI/SNF subunit gene, mutations affect specific residues or structural/functional domains. Data from the exome studies analyzed here did not reveal obvious mutation “hotspots” (Figure S1). However, the data are too sparse to draw firm conclusions. We note that a few validation studies, evaluating single SWI/SNF subunits (e.g. *ARID1A*) in much larger cohorts, have also not observed mutation hotspots [13], [15]. In this respect, SWI/SNF appears to differ from *TP53*, where mutations disproportionally target a small number of codons, and mostly occur within a single (in this case, DNA-binding) domain [46].

**Figure 3:**
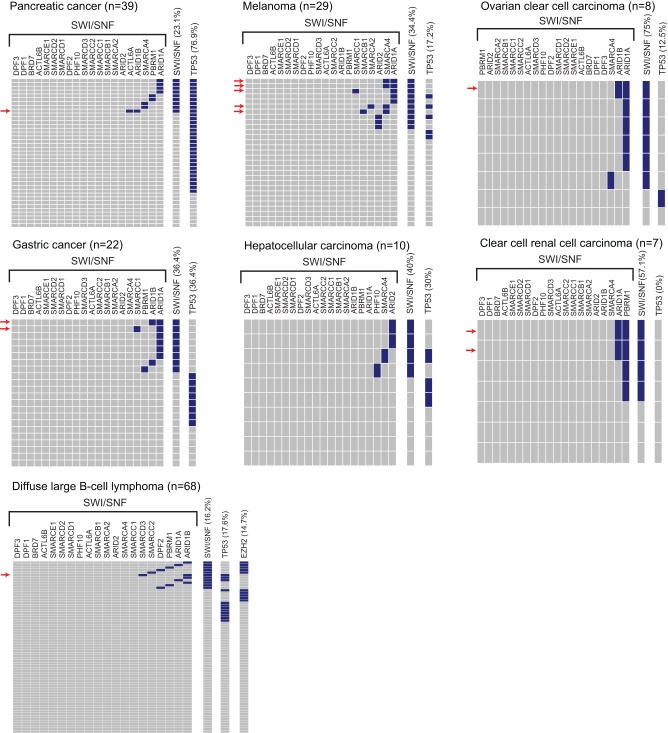
Co-occurrence of mutated SWI/SNF subunits. Heatmaps depict the mutation status of each SWI/SNF subunit gene in each tumor sample, shown for the seven tumor types with the highest frequency of SWI/SNF mutations. Rows and columns represent tumor samples and SWI/SNF subunit genes, respectively. Blue indicates the presence of a nonsynonymous mutation. Samples with mutations in two different SWI/SNF subunits are identified by a red arrow. *TP53* mutations are also indicated, as are *EZH2* activating mutations for the DLBCL study (*lower left* panel).

We also investigated, across the cancer types, whether mutations of different SWI/SNF subunits were mutually-exclusive of one another. Somewhat surprisingly, mutations in two different SWI/SNF subunits occurred within the same patient tumor about as often as would be expected by chance (i.e. as estimated by the square of SWI/SNF mutation frequency in a given tumor type) (Fig. 3). This finding suggests that mutational hits in two different SWI/SNF subunits are not functionally redundant, but rather that each might provide incremental perturbation or disruption of the complex.

### SWI/SNF mutations are not mutually-exclusive of other cancer gene mutations

The tumor suppressive function of SWI/SNF has been proposed to operate by controlling the expression or activity of specific genes and pathways, including Rb, TP53, Polycomb, sonic hedgehog, Myc, stem cell programs, and nuclear hormone receptor signaling [3], [47]. Our exome analysis provided a unique opportunity to try to systematically identify the key pathways mediating SWI/SNF tumor suppression, by mutual exclusivity analysis. Specifically, two different genes operating along in the same linear pathway, e.g. *KRAS* and *BRAF*, are thought less likely to be mutated in the same tumor sample because the mutations would be functionally redundant. Thus, identifying cancer genes that are mutated only in tumors without SWI/SNF mutation would imply a shared pathway. Likewise, identifying cancer genes that are always mutated in tumors with SWI/SNF mutation (mutually inclusivity) might suggest necessary cooperating pathways.

To address mutual exclusivity, we first focused on the relation between SWI/SNF and mutations of *TP53*. Recent studies reported mutual exclusivity of *ARID1A* and *TP53* mutations in both ovarian clear cell carcinoma and gastric cancer [13], [47]. Our analysis of exome datasets affirmed a mutually-exclusive relationship between SWI/SNF and *TP53* mutations in ovarian clear cell carcinoma and gastric cancer (Fig. 3); though statistical significance was only reached for gastric cancer (P = 0.018; Fisher's exact test). Notably, however, no such mutually exclusive relationship was apparent for other tumor types, including pancreatic cancer, melanoma, hepatocellular carcinoma, and DLBCL (Fig. 3). Indeed, in pancreatic cancer, all cases with SWI/SNF mutations actually carried *TP53* mutations, suggesting a trend towards mutual inclusivity (*P* = 0.085; Fisher's exact test).

Furthermore, the data suggest a need for caution in interpreting the mutual-exclusivity of SWI/SNF and *TP53* mutations. Ovarian and gastric cancers are both histological and genetically diverse diseases, and mutual exclusivity may rather correlate with tumor subtypes rather than reflect a mechanistic relation. Indeed, in gastric cancer SWI/SNF mutations tend to occur in MSI tumors, whereas *TP53* mutations generally occur in microsatellite stable tumors [13]. Thus, mutual exclusivity here may relate more to distinct mutagenic processes.

SWI/SNF has also been proposed to suppress tumor growth by antagonizing the oncogenic effects of polycomb repressive complex 2 (PRC2), mirroring its role in development [14], [48]. Approximately 15% of DLBCL cases harbor activating mutations of *EZH2*, the enzymatic component of PRC2. Thus, DLBCL provides an opportunity to assess the mutual exclusivity of SWI/SNF and PRC2 alterations. Notably, our analysis revealed several patient samples having both SWI/SNF and *EZH2* mutations (Fig. 3), not supportive of mutual exclusivity.

**Figure 4:**
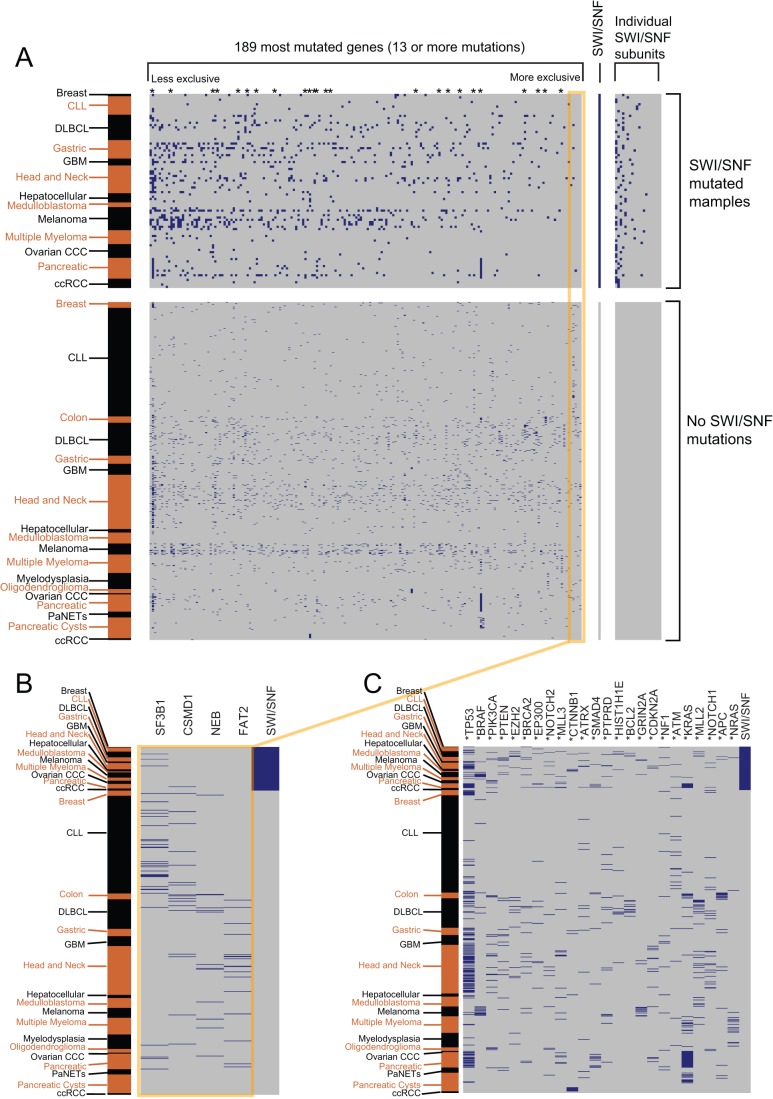
SWI/SNF mutations are not mutually exclusive of mutations in other commonly mutated genes. For each panel, rows correspond to tumor samples and columns correspond to genes. Within the matrices, blue corresponds to a nonsynonymous mutation while grey corresponds to no reported mutation. The rows are ordered first based on the SWI/SNF mutational status and second on the cancer subtype (annotated in alternating black and brown text, *left*). **A.** The mutational status of the 189 most-highly mutated genes across the exome studies, in relation to SWI/SNF mutational status. The 189 genes are rank-ordered from left to right, from those most mutationally-inclusive to those most mutationally-exclusive with SWI/SNF mutations. **B.** Zoomed-in view of the mutational status of the four most-exclusive gene mutations (*FAT2*, *NEB*, *CSMD1*, *SF3B1*); none reach statistical significance. Additional discussion is provided in Text S1. **C.** Zoomed-in view of the mutational status of select cancer genes. These genes are denoted by an asterisk in panel *A*. Additional discussion is provided in Text S1.

We next sought to take a more systematic approach to identify cancer gene mutations exhibiting mutual exclusivity with SWI/SNF mutation. To this effect, we analyzed the top 189 mutated genes (all genes with ≥13 mutations) across the 24 exome studies. The 189 genes included other well-known cancer genes (e.g. *KRAS*, *BRAF*, *CDKN2A*, *PTEN*, *NF1*, *APC*, *SMAD4*, etc) and represented many of the canonical signaling pathways (e.g. Ras, PI3K, Wnt, Notch, etc) in cancer. In spite of this, no significant mutually-exclusive (or mutually inclusive) relationships with SWI/SNF mutations were identified (Fig. 4 and Text S1).

It is possible that our analysis was under-powered to identify true mutually-exclusive relationships. Alternatively, it is possible (and we favor the explanation that) SWI/SNF rather effectuates tumor suppression by impacting multiple pathways, including Rb, TP53, Polycomb, sonic hedgehog, Myc, stem cell programs, nuclear hormone receptor signaling, and likely others that remain to be discovered. This “one-to-many” relationship would obscure mutual-exclusivity analysis. For example, mutations might occur in both PRC2 (*EZH2*) and SWI/SNF because they occur first in PRC2 and the later-occurring SWI/SNF mutation also impacts other pathways, or because the SWI/SNF mutation only partially activates PRC2 (which is further activated by *EZH2* mutation).

Several lines of evidence support the multi-functioning of SWI/SNF in tumor suppression. First, SWI/SNF mutations occur across tumors from diverse tissue types, suggesting they impact one or more broadly conserved processes. Second, recent studies seeking to map the genome binding sites of SWI/SNF (for example by chromatin-immunoprecipitation and deep-sequencing) have revealed on the order of 5,000–10,000 binding sites [49], [50], and thus SWI/SNF likely impacts many genes and pathways. Finally, it has long been known that SWI/SNF controls diverse biological processes (e.g. mating type switching and sugar fermentation in yeast), and it is thus reasonable to think that the same might be true of relevant processes (e.g. growth, survival, metabolism) in cancer cells.

### Conclusions

Our analysis of exome datasets represents the first systematic study of SWI/SNF mutations across human cancer. We find that SWI/SNF mutations are ubiquitous across diverse cancer types, and that functionally deleterious mutations predominate, supporting their being cancer driving events. Mutations preferentially hit certain key enzymatic and targeting subunits, implying that they serve to disrupt SWI/SNF complex activity. Finally, our inability to identify cancer gene mutations that are mutual exclusive of SWI/SNF mutations suggests that SWI/SNF likely impacts multiple cancer-relevant genes and pathways. Our analysis provides a rich resource for future mechanistic studies of the biological roles of SWI/SNF in human cancer.

## Methods

### Exome datasets

Exome datasets were identified from PubMed search up to April 30, 2012. We included studies having at least 5 samples, and publicly-accessible data. Datasets included are listed in Table 1. Annotated mutation data were retrieved, and parsed using custom Perl scripts.

### Determining mutational frequencies

Whole-exome mutational frequencies (as reported in Fig. 1B) were determined as the median levels across all studies. The median ratio of nonsynonymous mutations to synonymous mutations across studies was 2.95:1 (refer to Table S1 for exome-wide mutational summaries across each study), which corresponds to 25.3% synonymous mutations and 74.7% nonsynonymous mutations. Studies that did not report the frequency of synonymous mutations were not included in the median calculation. The same approach was used to breakdown the nonsynonymous mutations between missense mutations and other more damaging mutations, and to breakdown the missense mutations as damaging or benign. Here, damaging mutations refer to those called as “damaging” by Polyphen-2 or “intolerated” by SIFT. Benign mutations are those called as “possibly damaging” and “benign” mutations by Polyphen-2 or “tolerated” mutations by SIFT.

For SWI/SNF mutations, the ratio of nonsynonymous to synonymous mutations was determined by analyzing only those studies that reported synonymous mutations. After determining the frequency of synonymous mutations, the nonsynonymous mutations were broken down by their frequencies across all studies (all studies included this information). To breakdown missense mutations, the predicted functional consequence of each missense mutation was used. For the studies that did not report this information, we manually determined the predicted consequences using Polyphen-2. All of these data are summarized in Table S2. While there was some variation among the exome studies, the relative ratio of mutational types (both exome-wide and within SWI/SNF) was similar.

### Statistical analysis

A skew of SWI/SNF mutation classes (Fig. 1B) was evaluated by the chi-square test, with the expected distribution estimated from the whole-exome mutation data. Mutual exclusivity was assessed by two-sided Fisher's exact test. *P*-values of less than 0.05 were considered statistically significant.

## Supporting Information

Figure S1 **Distribution of mutations within the four most commonly mutated SWI/SNF subunit genes,** ***ARID1A*** **,** ***ARID1B*** **,** ***PBRM1*** **and** ***SMARCA4*** **.** Frequency plots show the number of mutations (identified from the 24 exome studies) at each codon position of *ARID1A* (NM_006015), *ARID1B* (ENST00000275248), *PBRM1* (NM_181042), and *SMARCA4* (NM_003072). Mutations are annotated as follows: * = nonsense; fs = frame shift; ss = splice site; bp = breakpoint. Protein domains are indicated, abbreviated as follows: ARID (ARID (A/T-rich interaction domain)/BRIGHT DNA binding domain); Bromo (Bromodomain, polybromo repeat); BAH (Bromo Adjacent Homology domain); HMG (High Mobility Group box); HSA (domain in helicases and associated with SANT domains); BRK (domain in transcription and CHROMO domain helicases); DEXDc (DEAD-like helicases superfamily); HELICc (Helicase superfamily c-terminal domain); Bromo SNF2L2 (Bromodomain, SNF2L2-like subfamily).
(PDF)

Table S1 Genome-wide mutational characteristics.
(XLS)

Table S2 Canonical SWI/SNF subunit mutational status in each study.
(XLS)

Text S1 **Additional discussion of** **Fig. 4** **.**
(DOC)
