# Supplementary figures and images for: The Spectrum of SWI/SNF Mutations, Ubiquitous in Human Cancers
Source: PLoS One. 2013 Jan 23;8(1):e55119. doi: 10.1371/journal.pone.0055119 (PMC3552954; doi:10.1371/journal.pone.0055119)

Figure S1

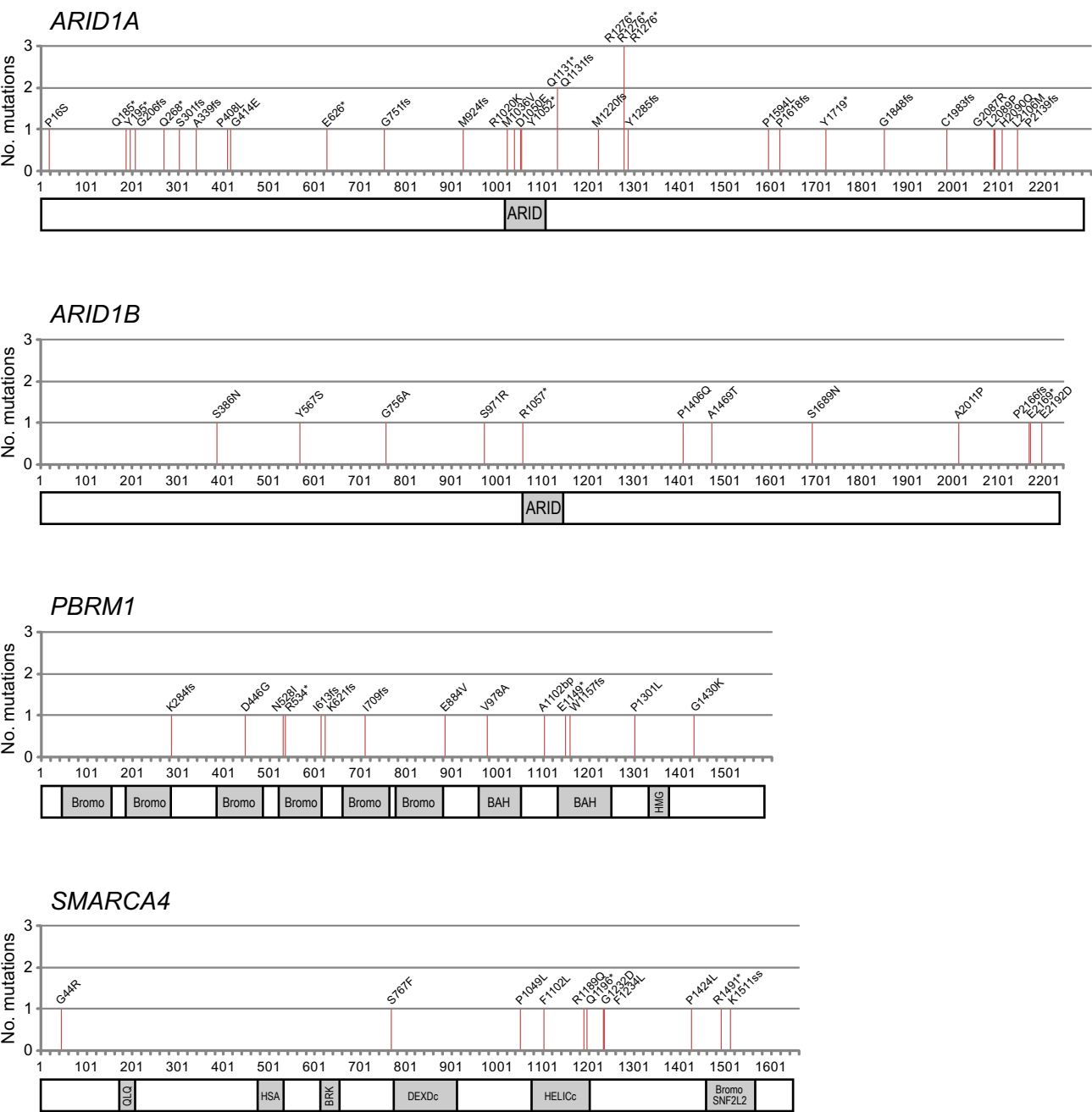

Supplement: Figure S1 — Distribution of mutations within the four most commonly mutated SWI/SNF subunit genes, ARID1A , ARID1B , PBRM1 and SMARCA4 . Frequency plots show the number of mutations (identified from the 24 exome studies) at each codon position of ARID1A (NM_006015), ARID1B (ENST00000275248), PBRM1 (NM_181042), and SMARCA4 (NM_003072). Mutations are annotated as follows: * = nonsense; fs = frame shift; ss = splice site; bp = breakpoint. Protein domains are indicated, abbreviated as follows: ARID (ARID (A/T-rich interaction domain)/BRIGHT DNA binding domain); Bromo (Bromodomain, polybromo repeat); BAH (Bromo Adjacent Homology domain); HMG (High Mobility Group box); HSA (domain in helicases and associated with SANT domains); BRK (domain in transcription and CHROMO domain helicases); DEXDc (DEAD-like helicases superfamily); HELICc (Helicase superfamily c-terminal domain); Bromo SNF2L2 (Bromodomain, SNF2L2-like subfamily). (PDF) [file pone.0055119.s001.pdf]
