# Supplementary material for: The Spectrum of SWI/SNF Mutations, Ubiquitous in Human Cancers
Source: PLoS One. 2013 Jan 23;8(1):e55119. doi: 10.1371/journal.pone.0055119 (PMC3552954; doi:10.1371/journal.pone.0055119)
Supplement: Text S1 — Additional discussion of Fig. 4 . (DOC) [file pone.0055119.s004.doc]

**The spectrum of SWI/SNF mutations, ubiquitous in human cancers**

A. Hunter Shain and Jonathan R. Pollack

**TEXT S1**

Figure 4A depicts the mutational relationship between SWI/SNF and the 189 most-highly mutated genes across these 669 tumor samples. Each of these genes had at least 13 mutations. This cutoff was chosen because it becomes difficult to infer statistical significance with fewer than 13 mutations for a study of this size. Indeed, a threshold of 27 mutually-exclusive mutations is required to reach statistical significance (i.e., two-sided Fisher’s exact test with *P*<0.05). However, we also included genes with 13-26 mutations in order to evaluate possible (non-significant) trends.

Among these 189 genes, only 2 showed a complete mutually-exclusive mutational pattern with SWI/SNF, *FAT2* and *NEB* (Fig. 4B). *FAT2* and *NEB* had 17 and 13 total mutations respectively, such that they just made the cutoff to be included in this analysis. Using a Fisher’s exact test for significance, the *P*-value for this mutual exclusivity is 0.15 (*FAT2*) and 0.39 (*NEB*). These events could very likely have occurred by random chance, especially when accounting for multiple hypotheses tested (189 genes). Furthermore, the mutational patterns suggest that these may just be passenger mutations. Both genes are very large, giving them a large footprint to accrue mutations. *FAT2* encodes an integral membrane protein, a member of the cadherin-type repeat family, and is 479 KD. NEB encodes nebulin, a component of the cytoskeletal matrix in skeletal muscle, and is 773 KD. Nearly all mutations hitting each gene are benign missense mutations, and the more damaging mutations typical of tumor suppressor genes are not present. Based on these characteristics, we suspect that the mutations are passenger events that happen to be exclusive with SWI/SNF mutations in this cohort.

Beyond *FAT2* and *NEB*, no other genes showed purely mutually-exclusive mutational patterns with SWI/SNF, though some trended towards that direction. The two with the most significant trend in that direction were *CSMD1* and *SF3B1* (Fig. 4B). However, these genes were preferentially mutated in tumor subtypes where SWI/SNF mutations were either rare or nonexistent. Thus, the trends towards exclusivity are probably not driven by pathway epistasis but instead are correlated with tumor subtypes.

Using the unbiased search described above, we were unable to identify any genes with a significant or a compelling pattern of mutational exclusivity. Candidate genes generally fell into two categories – those which had mutational patterns characteristic of passenger mutations, and lineage-specific cancer genes from cancers subtypes that do not have high frequencies of SWI/SNF mutations. Even with 669 samples, this is likely still an underpowered analysis and perhaps future efforts with larger sample numbers might yield promising candidates.

In addition to an unbiased search, we also looked more closely at the mutational patterns of 24 previously known cancer genes that were frequently mutated in these datasets (Fig. 4C). These genes cross most of the canonical pathways in human cancer. First, *TP53* and *EZH2* are respectively nodes in the p53 and Polycomb cancer pathways – two pathways that have been implicated as downstream effectors of SWI/SNF. Mutations in these genes were not exclusive with SWI/SNF mutations across all tumors (Fig. 4C). These results and their implications are discussed further in the main text.

In addition to *TP53* and *EZH2*, several other cancer genes were examined. These included *KRAS*, *NRAS*, *NF1*, and *BRAF*, nodes in the RTK-Ras-MAPK signaling pathway, and *PIK3CA* and *PTEN*, nodes in the PI3K signaling pathway, and together among the most commonly mutated genes in all cancers. *BRCA2* and *ATM* encode caretaker proteins involved in genomic stability and DNA repair. *ATRX*, *EP300*, *MLL3*, and *MLL2* encode chromatin remodelers – like SWI/SNF. *APC* and *CTNNB1* are nodes in the Wnt/β-catenin signaling pathway, which is often activated in gastrointestinal tumor subtypes. *SMAD4* is a node in the TGFβ tumor suppressor pathway, a pathway that SWI/SNF has been biochemically implicated to mediate. *HIST1H1E* is a member of the linker histone, H1, family, and SWI/SNF is known to remodel histones. *BCL2* encodes an antiapoptotic protein. *CDKN2A* is a commonly lost tumor suppressor gene which acts in both the p53 and the Rb pathways. Lastly, *NOTCH1* and *NOTCH2* are nodes in the NOTCH signaling pathway, a well characterized pathway that controls proliferation in various contexts. Notably, SWI/SNF mutations were not mutually exclusive with any of the genes discussed above.

One interpretation of the coexistence in mutations targeting both SWI/SNF and all of the major cancer pathways above is that SWI/SNF does not act through any of these pathways, despite the reports linking SWI/SNF to several of these pathways. While possible, we think this is unlikely. As discussed further in the main text, we believe SWI/SNF may mediate multiple cancer pathways. In such a capacity, it would be possible for SWI/SNF mutations to coexist with any driver mutation, thus explaining our inability to identify a compelling pathway even in our unbiased analysis.
